# Control of nuclear envelope dynamics during acute ER stress by LINC complexes disassembly and selective, asymmetric autophagy of the outer nuclear membrane

**DOI:** 10.1080/15548627.2023.2299123

**Published:** 2023-12-28

**Authors:** Marika K. Kucińska, Maurizio Molinari

**Affiliations:** a Faculty of Biomedical Sciences, Institute for Research in Biomedicine, Università della Svizzera italiana (USI), Bellinzona, Switzerland; b Department of Biology, Swiss Federal Institute of Technology, Zurich, Switzerland; c School of Life Sciences, École Polytechnique Fédérale de Lausanne, Lausanne, Switzerland

**Keywords:** ER-phagy, ONM-phagy, ER stress, Nuclear envelope, KASH proteins, LINC complex

## Abstract

The endoplasmic reticulum (ER) extends to the outer (ONM) and the inner (INM) nuclear membrane forming the nuclear envelope (NE) that delimits the nucleoplasm containing the cell genome. Unfolded protein responses (UPRs) and reticulophagy responses increase or reduce ER size and activities, respectively. If dynamic changes of the ER are transmitted to the contiguous NE was not known. In our recent publication, we report on the transmission of stress-induced ER expansion to the NE, which requires disassembly of the Linker of Nucleoskeleton and Cytoskeleton (LINC) complexes deputed to ensure a physical connection between the cytoplasmic cytoskeleton and the nuclear lamina and to maintain the width between INM and ONM within 50 nm. LINC complexes disassembly relies on reduction of the disulfide bond that covalently links SUN proteins in the INM and KASH proteins (SYNE/NESPRIN proteins in mammals) in the ONM by the ONM-resident reductase TMX4. Upon stress resolution, the physiological shape of the NE is reestablished by SEC62-driven ONM-phagy, where ONM-derived vesicles are directly captured by RAB7- and LAMP1-positive endolysosomes in processes that proceed via asymmetric microautophagy of the NE.

## When cells are stressed, the ER gets bigger

The size and activity of the ER can rapidly be adapted to the cell’s needs. Physiological or pathological signals or stresses activate UPRs to increase the ER volume and fill the organelle lumen and membranes with biosynthetic enzymes, ion channels and pumps, molecular chaperones. Among physiological cues, the ER expansion upon differentiation of a B cell to a plasma cell that produces in the ER and secretes extracellularly more than 2000 immunoglobulin chains/sec is an amazing example of the magnitude of this phenomenon.

## When the stress is over, the ER resumes physiological size and activity

**Figure 1.**
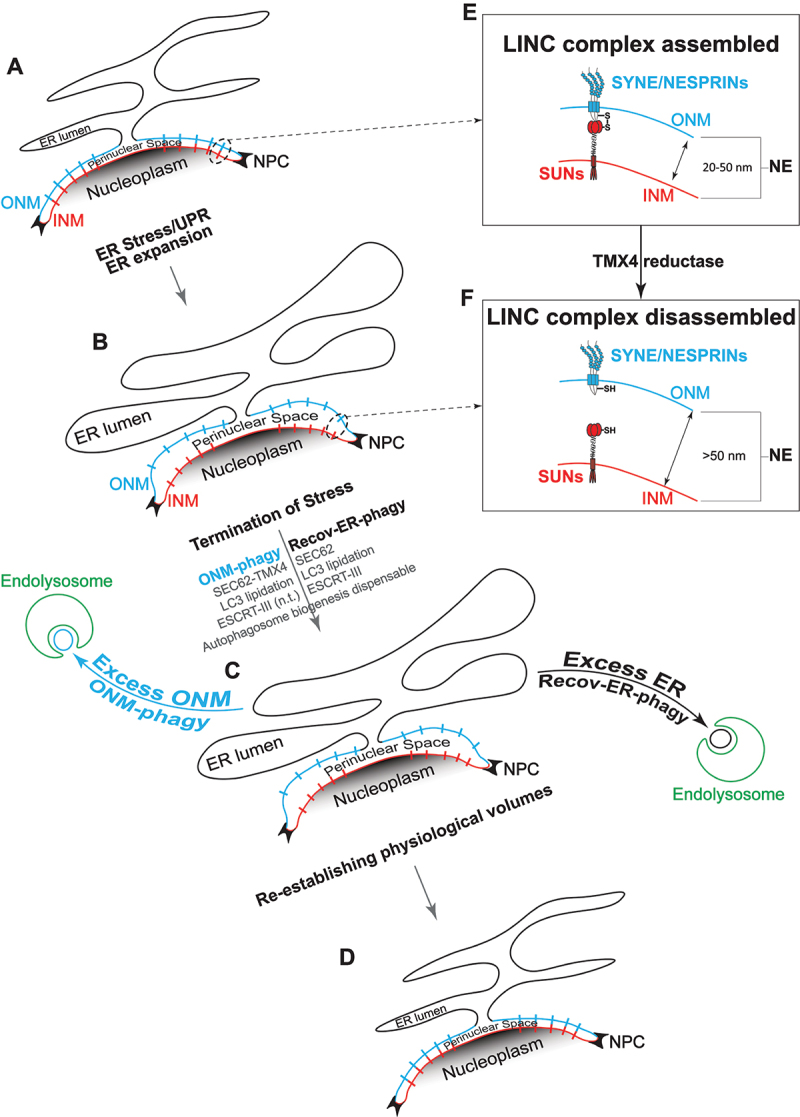
NE ultrastructure and dynamics. (A) representation of the ER with the membrane continuing in the ONM. The ER lumen communicates with the perinuclear space. Two nuclear pore complexes are shown (NPC). (B) upon perturbation of the ER homeostasis, both ER and NE swell, resulting in the expansion of the perinuclear space. (C) interruption of the stress signal activates autophagy programs (recov-ER-phagy and ONM-phagy) to restore physiological ER and NE shape. Involvement of SEC62, TMX4, LC3 lipidation and ESCRT-III is shown below the arrows. (D) the pre-stress size and function of ER and NE is restored after few hours. (E) magnification of an assembled LINC complex. (F) magnification of a disassembled LINC complex. n.T.: not tested.

In some cases, as illustrated for the differentiation of a B cell to a plasma cell, the expansion of the ER is an irreversible process. Antibody-producing plasma cells eventually die after a few days of fight against the invading pathogen. However, there are documented examples of ER expansion followed by lysosomal clearance of the excess ER generated during the UPR phase to resume the physiological size of the compartment. The group of Weibel reported in the late 1960s that treatment of rats with phenobarbital, an anti-epileptic drug, causes swelling of the ER in liver hepatocytes (Figure 1A, B). A few years later, the same group made the seminal observation that upon interruption of the pharmacological treatment, the ER resumes its original size (Figure 1B–D). Weibel and friends observed that during the recovery phase, the ER is fragmented, and ER portions are selectively eaten-up by single membrane, degradative, lysosomal compartments. This is one of the first reports on selective reticulophagy, which had to await for mechanistic dissection several decades.

## SEC62 and the process of recov-ER-phagy

Cyclopiazonic acid proved optimal to induce a UPR that was rapidly shut down upon wash-out of this reversible inhibitor of the sarcoplasmic/endoplasmic reticulum Ca^2+^-ATPase. Our study revealed the involvement of the translocon component SEC62 as the LC3-binding reticulophagy receptor that regulates lysosomal clearance of excess ER during recovery from ER stress (recov-ER-phagy). Upon ER fragmentation, the excess ER portions are directly engulfed by RAB7- and LAMP1-positive endolysosomes under the control of the ESCRT-III machinery (Figures. 1C, black arrow, recov-ER-phagy). Thus, piecemeal microreticulophagy mechanistically defines the resumption of physiological ER size and activity upon ER stress resolution.

## And the nuclear envelope?

The ER membrane (black in Figure 1) continues in the ONM (blue) that merges at the nuclear pore complexes (NPC) with the INM (red). Consequently, the ER lumen is contiguous to the perinuclear space enclosed between ONM and INM (Figure 1). In our latest publication [1], inspection of room temperature and cryo-electron microscopy micrographs reveals that in mammalian cultured cells, the expansion of the ER induced by cyclopiazonic acid is transmitted to the NE. The distance between the ONM and the INM increases from 20–50 nm measured at steady state, to up to 180 nm, with a considerable expansion of the perinuclear space. The physiological NE ultrastructure is restored a few hours after termination of the stress condition. Live cell imaging and electron tomography reveal that during recovery, the ONM vesiculates and ONM portions are engulfed by RAB7- and LAMP1-positive endolysosomes.

## Keeping the distance, breaking the distance: the dynamics of LINC complexes

The distance between the INM and the ONM is maintained between 25 and 50 nm by the LINC complexes. These are formed by INM proteins of the SUN family and ONM proteins of the SYNE/NESPRIN family, covalently associated via an intermolecular disulfide bond (Figure 1E). Transmission of the ER-expansion to the NE and the substantial increase of the distance between INM and ONM implies the rupture of the LINC complexes, which we verified by cryogenic Focus Ion Beam milling followed by cryo-electron tomography (FIB-CET) of vitrified cells.

## Reductive disassembly of the LINC complex by the ONM-resident reductase TMX4

Dissociation of SUN proteins from SYNE/NESPRIN partners in the LINC complex (Figure 1E, F) requires the intervention of a reductase that nucleophilically attacks the cysteine residue of one disulfide-bonded partner, thus releasing the other. Ectopic expression of trapping forms of redox enzymes carrying a point mutation in the catalytic site that prevents release of clients, immunoisolation of the complexes and identification of the clients by liquid chromatography with tandem mass spectrometry (LC-MS/MS) revealed endogenous SYNE/NESPRIN proteins as preferential clients of the reductase TMX4, an ill-defined member of the protein disulfide isomerase superfamily that we localized in the ONM by transmission immunoelectron microscopy.

## All in all: recov-ER-phagy and ONM-phagy

As we previously reported for the removal of excess ER to restore physiological size and activity during recovery from ER stress (Figure 1C, black arrow, recov-ER-phagy, 1D), also the return of the NE at physiological shape (Figure 1C, blue arrow, ONM-phagy, 1D) is driven by the activation of the reticulophagy receptor SEC62 and proceeds via microautophagy, where endolysosomes directly engulf the portions of ONM to be removed from cells (Figures. 1C). Silencing or deletion of TMX4 substantially inhibits the transmission of the ER swelling to the NE upon perturbation of ER homeostasis and the lysosomal clearance of ONM portions during the phase of recovery from acute stresses (Figure 1C, blue arrow).

## Outlook

Deletion of *Tmx4* is toxic to cells, and we observe that cells lacking TMX4 have a rigid, condensed NE, which does not swell upon ER perturbation. In mammalian cells, the perinuclear space must somewhat be expanded (i.e., the distance between the INM and the ONM must increase) for nuclear egress of ribonucleoprotein particles, misfolded protein aggregates and herpes virus capsids that cannot be transported from the nucleoplasm to the cytoplasm via the NPC. These macrostructures transit through the perinuclear space between INM and ONM on their way to the cytoplasm. The NE and the LINC complexes are also remodeled in cells exposed to pharmacological or mechanical stresses, during cell division, cell migration, etc. We postulate that redox-driven control of LINC complexes assembly may play a crucial role in these events.
